# Snord116 is critical in the regulation of food intake and body weight

**DOI:** 10.1038/srep18614

**Published:** 2016-01-04

**Authors:** Yue Qi, Louise Purtell, Melissa Fu, Nicola J. Lee, Julia Aepler, Lei Zhang, Kim Loh, Ronaldo F. Enriquez, Paul A. Baldock, Sergei Zolotukhin, Lesley V. Campbell, Herbert Herzog

**Affiliations:** 1 Neuroscience Division, Garvan Institute of Medical Research, St Vincent’s Hospital, Darlinghurst, NSW, Australia; 2 Diabetes and Metabolism Division, Garvan Institute of Medical Research, St Vincent’s Hospital, Darlinghurst, NSW, Australia; 3 Bone Biology Division, Garvan Institute of Medical Research, St Vincent’s Hospital, Darlinghurst, NSW, Australia; 4 Department of Pediatrics, College of Medicine, Center for Smell and Taste, University of Florida, Gainesville, Florida 32610, USA; 5 School of Medical Sciences, University of NSW, NSW, Australia

## Abstract

Prader-Willi syndrome (PWS) is the predominant genetic cause of obesity in humans. Recent clinical reports have suggested that micro-deletion of the Snord116 gene cluster can lead to PWS, however, the extent of the contributions of the encoded snoRNAs is unknown. Here we show that mice lacking Snord116 globally have low birth weight, increased body weight gain, energy expenditure and hyperphagia. Consistent with this, microarray analysis of hypothalamic gene expression revealed a significant alteration in feeding related pathways that was also confirmed by *in situ* hybridisation. Importantly, selective deletion of Snord116 only from NPY expressing neurons mimics almost exactly the global deletion phenotype including the persistent low birth weight, increased body weight gain in early adulthood, increased energy expenditure and hyperphagia. Mechanistically, the lack of Snord116 in NPY neurons leads to the upregulation of NPY mRNA consistent with the hyperphagic phenotype and suggests a critical role of Snord116 in the control of NPY neuronal functions that might be dysregulated in PWS.

Prader-Willi syndrome (PWS) is a paternally imprinted disorder that leads to developmental delay and genetic obesity^1^,^2^. The clinical symptoms of PWS patients present distinct biphasic metabolic stages. The first begins at birth or even prenatally and comprises low birth weight, severe neonatal hypotonia, developmental delay and failure to thrive. The later stage, with an age of onset between 2 and 6, is characterised by hyperphagia, which when not controlled leads to severe obesity during childhood and adulthood, as well as numerous other behavioural abnormalities including tantrums, learning disabilities and compulsive behaviour. PWS is a lifelong condition and, to date, there is no cure.

In humans, the critical region for PWS is located on paternal chromosome 15 (q11.2-13). The three types of genetic defect causing PWS are non-inherited deletions of expression (70%), maternal uniparental disomy 15 (20–25%) and genomic imprinting defects (2–5%)^2^,^3^,^4^,^5^. The PWS locus contains several known protein coding genes (MKRN3, MAGEL2, NECDIN, C15ORF2, and SNURF-SNRPN (small nuclear ribonucleoprotein N)) as well as non-coding genes. The gene encoding for SNURF-SNRPN was initially considered as a strong candidate for PWS, as patients with microdeletions of different intervals of SNRPN (a 847 kb deletion including the non-coding genes^6^ and exon 1 of SNRPN (a 35 kb deletion^7^, a 6 kb deletion^8^ and a 4.3 kb deletion^9^) exhibited a PWS-associated phenotype. Subsequent evidence from patients with balanced translocations, however, excluded it as a primary candidate behind the development of the syndrome^10^,^11^. Instead, these reports highlighted the importance of a family of small nucleolar RNAs (snoRNAs), located within the introns of the SNURF-SNRPN gene, in the development of PWS^4^,^10^,^11^,^12^,^13^,^14^.

Six snoRNAs have been reported within this location, which include SNORD64 (single copy), SNORD107 (single copy), SNORD108 (single copy), SNORD109 (two copies), SNORD116 (29 copies) and SNORD115 (48 copies)^14^,^15^. These belong to the C/D box class of non-coding snoRNAs, some members of which have been shown to perform site-specific 2′-O-methylation of rRNAs and spliceosomal small nuclear RNAs^16^. In addition, SNORD115 has been proposed to be involved in the regulation of alternative splicing of the serotonin receptor 2C pre-mRNA, potentially contributing to the development of obesity^17^. However, other studies in humans and mice have excluded it as a candidate gene for PWS^14^,^18^,^19^. Importantly, recent studies have identified several individuals who have microdeletions in the PWS locus that display many of the characteristics of PWS, who all have the absence of the SNORD116 cluster in common^13^,^14^,^20^,^21^,^22^.

The PWS locus in mice is highly homologous to that of humans, with the difference that the Snord116 cluster in mice contains 40 tandem copies and is expressed exclusively in the brain. Mice with deletions encompassing the Snord116 gene cluster have been generated, including one in which the deletion removes the Snrpn coding transcript and all snoRNAs between Snrpn and Ube3a but leaves the imprinting centre intact^7^,^8^,^23^,^24^. These mutant mouse models suffer severe postnatal growth retardation and the high lethality rate (80% die before weaning) and the multi-deletion of genes make phenotypic characterisation of mature mice difficult. A single-gene knockout model, the Snord116 deletion mouse, has recently been generated^21^. These mice, with paternally inherited Snord116 deletions, survive to adulthood and exhibit a subset of classical clinical phenotypes of PWS but further studies are required to explain the underlying mechanism caused by the gene deletion.

The distribution of Snord116 expression in the hypothalamus overlaps strongly with appetite regulating centres^25^ and a hyper-responsive food-related reward circuit has also been shown in PWS patients^26^. The two major populations of neurons that control appetite and energy homeostasis regulation are the orexigenic acting neuropeptide Y (NPY)/agouti-related peptide (AgRP) neurons and the anorexic proopiomelanocortin (POMC)/cocaine and amphetamine regulated transcript (CART) neurons located in the arcuate nucleus of the hypothalamus (Arc). Dysregulation in expression profiles of these two types of neurons are commonly seen in genetic and diet-induced obesity models such as the leptin deficient ob/ob mice or high fat diet (HFD) fed mice, respectively. However, only limited information is available for the levels of expression in human PWS, and therefore the functionality of these central circuitries in PWS remains unclear.

Snord116 is a paternally imprinted gene, which signifies that the resultant phenotype of this gene depends on the parent-of-origin. Thus most studies of genomic imprinting in PWS have assumed a simple pattern of imprinting, where the paternally inherited copy is expressed while the maternal copy is silenced^27^. However, a quantitative study on the effects of an imprinted allele, specifically on the genes in chromosome 7 in mice, indicated that more complex patterns of imprinted genes exist which depend on both parents, and that the effects of imprinted genes were stronger at later stages of development^28^. Therefore, to investigate the phenotype resulting from global Snord116 deletion more comprehensively, instead of using heterozygous deletion mice^21^, a biallelic Snord116 deletion model was used in this study. Two selected time intervals were investigated to identify early and late stage effects. In addition, to more specifically investigate the influence of Snord116 on the control of the appetite stimulating circuits initiated by the NPY system we also generated a conditional knockout removing Snord116 only in NPY neurons and tested the consequences of this disruption on whole body energy homeostasis regulation. Male and female animals were investigated; however, since results were almost identical between genders and for reasons of clarity and space only data from male mice are shown in the main text and female data are provided as supplementary information.

## Results

### Low body weight and altered body composition in mice lacking Snord116

**Figure 1:**
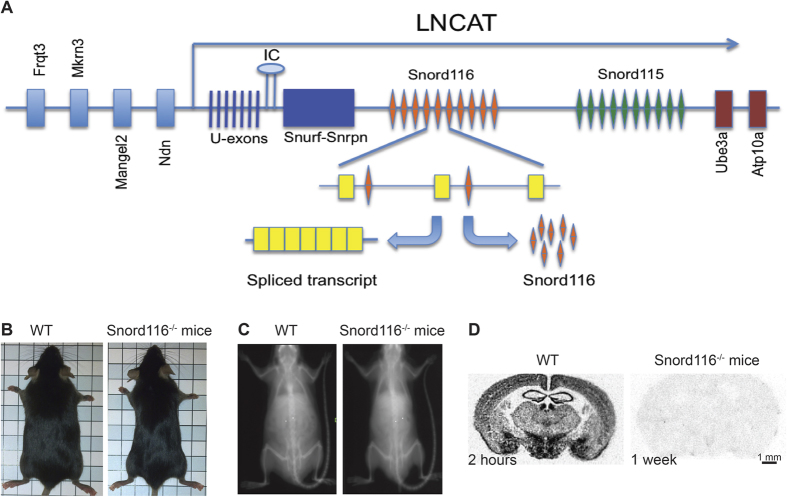
Generation of Snord116^−/−^ mice. (**A**) Schematic representation showing the genomic organisation of the PWS-related genes in the critical region of chromosome 15q 11.2-13 and the transcription of the Snord116 cluster. (**B**) Representative photos of male control mice (left) and gender-matched Snord116^−/−^ mice (right) at 12 weeks of age. Each square represents 1x1 cm^2^. (**C**) Images of dual energy x-ray (DXA) absorptiometry of the control (left) and Snord116^−/−^ mouse (right). (**D**) The scanned images of *in situ* hybridisation experiments showing the expression of Snord116 in coronal sections of mouse brains through the hypothalamus and amygdala. The films were exposed to the radioactive sections for 2 hours for control mice (left) and 1 week for Snord116^−/−^ mice (right).

In order to determine the phenotype caused by the germline deletion of the Snord116 cluster (Fig. 1A) in homozygous mice, floxed Snord116 mice (Snord116^lox/lox^) (Ding, Li *et al.* 2008) were crossed with a germline oocyte-specific Cre line and the resultant heterozygous Snord116 knockout mice (Snord116^+/−^) were crossed to generate Snord116^−/−^, Snord116^+/−^ and Snord116^+/+^ control mice (Fig. 1B,C). Only Snord116^−/−^ and Snord116^+/+^ control mice without the Cre-recombinase transgene were used in the analysis. The successful deletion of the Snord116 gene was then confirmed by *in situ* hybridisation of brain sections from Snord116^−/−^ mice and the controls (Fig. 1D).

**Figure 2:**
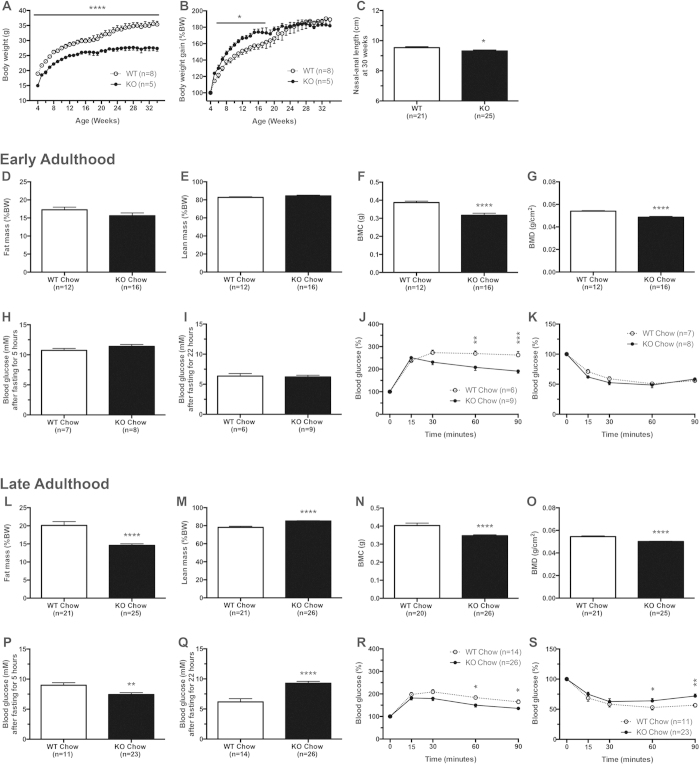
Low body weight, increased growth rate and altered body composition in male Snord116^−/−^ mice (—●—; black bars) vs. control mice (···O···; white bars) on chow diet. (**A**) Absolute weekly body weight from 4 to 34 weeks of age; (**B**) growth rate normalised to body weight (BW) at 4 weeks of age; (**C**) nasal-anal length. In early adulthood (12-16 weeks of age): (**D**) fat mass as a percentage of BW (%BW); (**E**) lean mass (%BW); (**F**) bone mineral content (BMC); (**G**) bone mineral density (BMD); (**H**) blood glucose levels after 5 hours fasting; (**I**) blood glucose levels after 22 hours fasting; (**J**) blood glucose curves during intraperitoneal glucose tolerance test as a percentage of fasting glucose levels; (**K**) blood glucose curves during insulin tolerance test as a percentage of fasting glucose levels. In late adulthood (28–32 weeks of age): (**L**) fat mass as a percentage of BW (%BW); (**M**) lean mass (%BW); (**N**) bone mineral content (BMC); (**O**) bone mineral density (BMD); (**P**) blood glucose levels after 5 hours fasting; (**Q**) blood glucose levels after 22 hours fasting; (**R**) blood glucose curves during intraperitoneal glucose tolerance test as a percentage of fasting glucose levels; (**S**) blood glucose curves during insulin tolerance test as a percentage of fasting glucose levels. (*P < 0.05, **P < 0.01, ***P < 0.001, and ****P < 0.0001).

Male Snord116^−/−^ mice and their controls were then investigated by a multi-measure metabolic profiling procedure at two selective time intervals, age 12 to 16 weeks and age 28 to 34 weeks, to identify potential differences in the early and late stages of adulthood, respectively. As shown in Fig. 2A, analysed by two-way ANOVA, Snord116^−/−^ mice displayed low birth weight and this reduced weight persisted to early and late adulthood (P < 0.0001, F = 12.32). Importantly, however, body weight gain of Snord116^−/−^ mice in the first 18 weeks of life was actually increased (P < 0.05) (Fig. 2B) mimicking a similar phenotype to that seen in human PWS. At an older age however, this increased body weight gain was no longer seen and Snord116^−/−^ mice at 34 weeks of age actually weighed 27.4 ± 0.9 g compared to 35.4 ± 1.0 g in controls. This reduced body weight was also reflected in a shorter nasal-anal length in the Snord116^−/−^ mice, statistically analysed by t-test (P < 0.05) (Fig. 2C).

The lower body weight in the older Snord116^−/−^ mice was associated with a significantly reduced fat mass but increased lean mass as percentage of body weight (determined by dual energy X-ray (DXA) and analysed by t-test (P < 0.0001)) (Fig. 2D,E,L,M) as well as a significantly reduced bone mass in both young and old mice (analysed by t-test and P < 0.0001) (Fig. 2F,G,N,O). These DXA data are supported by the significant alterations in weights of dissected white adipose tissue (WAT) depots from different regions (data not shown). Similar results were obtained for female mice (Supplementary Figure 1 and 2).

### Germline deletion of Snord116 in mice improves glucose metabolism

One characteristic of patients with PWS often described is higher insulin sensitivity and lower insulin resistance^29^, therefore we investigated whether Snord116 deletion has an effect on insulin signalling. Whole blood glucose levels were not altered significantly in male Snord116^−/−^ mice compared to controls after fasting for either 5 hours or 22 hours in early adulthood (Fig. 2H,I). However, this changed in late adulthood with Snord116^−/−^ mice showing reduced glucose levels after 5 hours fasting (analysed by t-test and P < 0.01; Fig. 2P) and increased levels of glucose after 22 hours fasting (analysed by t-test and P < 0.0001; Fig. 2Q). During a glucose tolerance test (GTT), data analysed by two-way ANOVA with Bonferroni post-tests showed that blood glucose levels of Snord116^−/−^ mice were significantly reduced at 60 and 90 minutes compared to controls, indicating a significant improvement in glucose tolerance (P < 0.05, F = 8.500 for Fig. 2J and P < 0.05, F = 8.997 for Fig. 2R). Consistent with this, insulin tolerance tests (ITT) revealed a better response to exogenous insulin injection at the 60 and 90 min time points in Snord116^−/−^ mice specifically in late adulthood (Fig. 2S). An almost identical phenotype for these parameters was observed for female mice (Supplementary Figure 1).

### Increased food intake in mice lacking Snord116

**Figure 3:**
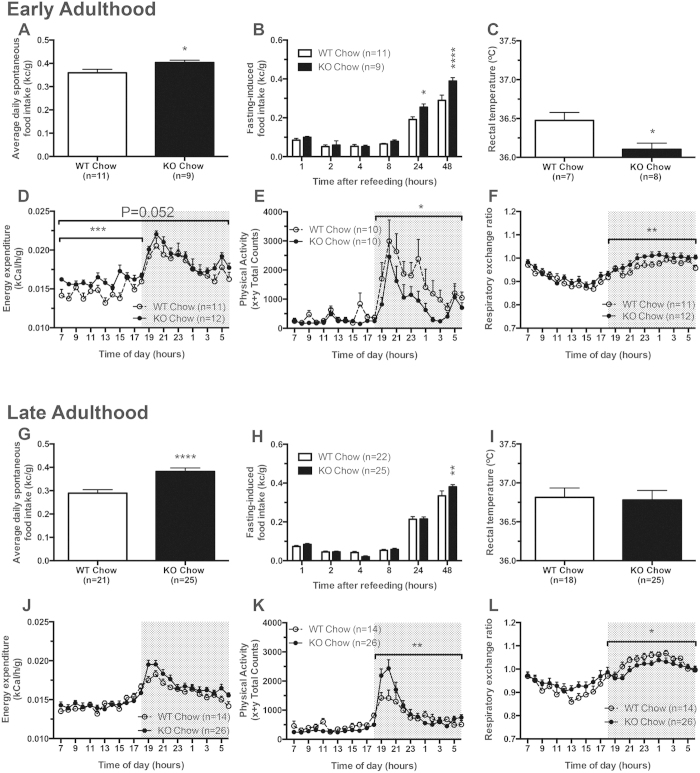
Increased energy intake and energy expenditure in male Snord116^−/−^ mice (—●—; black bars) vs. control mice (···O···; white bars) on chow diet. In early adulthood (12–16 weeks of age): (**A**) spontaneous 24-hour calorie intake as a percentage of body weight (%BW), expressed as the average of triplicate readings over three consecutive days; (**B**) fasting-induced calorie intake (%BW); (**C**) core body temperature; (**D**) 24-hour energy expenditure; (**E**) 24-hour physical activity; (**F**) 24-hour respiratory exchange ratio (RER). In late adulthood (28–32 weeks of age): (**G**) spontaneous 24-hour calorie intake as a percentage of body weight (%BW), expressed as the average of triplicate readings over three consecutive days; (**H**) fasting-induced calorie intake (%BW); (**I**) core body temperature; (**J**) 24-hour energy expenditure; (**K**) 24-hour physical activity; (**L**) 24-hour respiratory exchange ratio (RER). (*P < 0.05, **P < 0.01, ***P < 0.001, and ****P < 0.0001).

The most dramatic feature of individuals with PWS is the extreme hyperphagia. Interestingly, despite the reduced body weight of male Snord116^−/−^ mice, significant increase in spontaneous food intake analysed by t-test was observed in these mice. In early adulthood, control mice consumed, when normalised to body weight, 0.36 ± 0.02 kcal/g and Snord116^−/−^ mice consumed 0.40 ± 0.01 kcal/g of chow diet (analysed by t-test and P < 0.05) (Fig. 3A). In late adulthood, control mice reduced their food intake (0.29 ± 0.02 kcal/g) while Snord116^−/−^ mice still ate a similar amount of chow compared to when they were young (0.38 ± 0.02 kcal/g), widening the gap in food intake between the Snord116^−/−^ mice and the control mice even further (analysed by t-test and P < 0.0001) (Fig. 3G). Consistent with this, fasting-induced food intake was also increased in Snord116^−/−^ mice at the 24 and 48 hour time points, which was analysed by two-way ANOVA (P < 0.01 and F = 12.54) (Fig. 3B,H), accompanied by a normal body weight regain over this period. Taken together, the overactive feeding behaviour seen in Snord116^−/−^ mice, albeit without an associated development of obesity, is similar to the hyperphagic phenotype observed in humans with PWS, supporting a role for Snord116 in the early development of the PWS phenotype. Consistent with the male data, food intake was also significantly upregulated in female Snord116^−/−^ mice (Supplementary Figure 1).

### Lack of Snord116 leads to increased energy expenditure

The increase in energy intake in male Snord116^−/−^ mice is inconsistent with the reduced body weight and body composition. In order to explain this discrepancy, core body temperature was monitored over three consecutive days and analysed by t-test. Indirect calorimetry was used to determine energy expenditure and physical activity and results were analysed by two-way ANOVA. In early adulthood, Snord116^−/−^ mice showed reduced core temperature (P < 0.05) (Fig. 3C) and reduced activity specifically in the dark phase (P < 0.05) (Fig. 3E). On the other hand, energy expenditure (analysed by ANCOVA using lean mass as a co-variant) showed a significant increase in the light phase (P < 0.001, F = 6.751) and a trend towards increased expenditure over 24 hours (P = 0.05, F = 2.208) (Fig. 3D). However, these differences were no longer present in older Snord116^−/−^ mice (Fig. 3I,J), which instead showed increased physical activity in the dark phase (P < 0.01, F = 5.516) (Fig. 3K), partly explaining the increased caloric intake. In addition to the alterations in food intake and energy expenditure, there were differences between Snord116^−/−^ mice and control mice in fuel source preference. As assessed by the respiratory exchange ratio (RER), Snord116^−/−^ mice used more carbohydrate in the dark phase in early adulthood (P < 0.01) and more fat in the dark phase in late adulthood (P < 0.05) (Fig. 3F,L). In agreement with the male data female also showed significantly increased energy expenditure again contributing to the lean phenotype (Supplementary Figure 1)

### Altered Expression of Hypothalamic Neuropeptides and Hormones

Since expression of Snord116 in mice is restricted to the brain and is highest in the hypothalamus where food intake and energy homeostasis are mainly controlled, we next investigated potential changes in the expression pattern of neurotransmitters known to be involved in these processes. For this we employed microarray analysis using RNA extracted from hypothalamic tissue blocks of Snord116^−/−^ and WT mice. A gene set enrichment analysis (GSEA) was performed on the microarray data in order to evaluate changes at the level of gene sets, thus avoiding the bias of a candidate gene approach. This GSEA analysis, using the collection of gene sets derived from the Biological Process Ontology, revealed that 85 gene sets were significantly altered in Snord116^−/−^ at a false discovery rate (FDR) of <25%. Importantly, it revealed that the gene sets of ‘feeding behaviour’ and ‘skeletal development’ were significantly altered in Snord116^−/−^ mice (Supplementary Table 1) consistent with similar phenotypes in human subjects with PWS.

Since alternative splicing has been suggested to be one of the functional consequences of Snord116 action, we also investigated the data collected from this gene chip to analyse potential differences in alternative splicing in the different transcripts. Although transcripts with altered splicing pattern could be identified, there was no consistent pattern that would allow these gene products into a pathway linked to the observed phenotypes.

**Figure 4:**
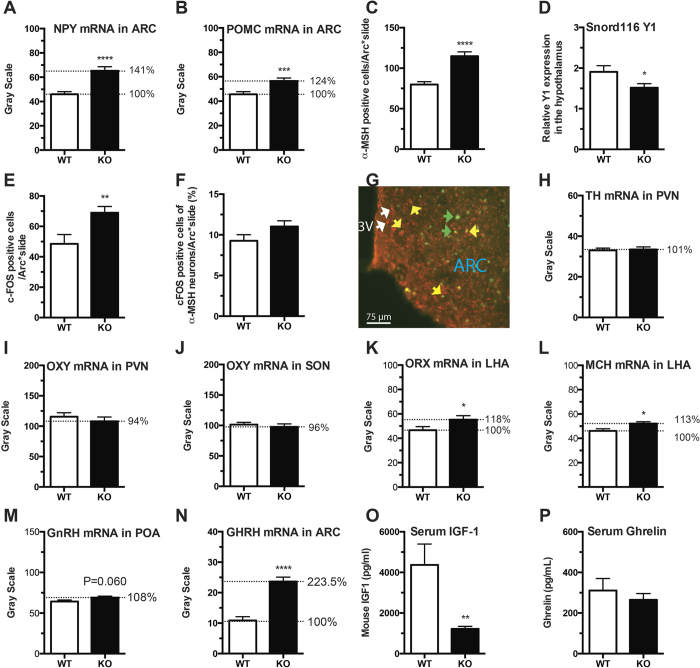
Altered hypothalamic-pituitary-growth hormone axis and increased expression of neuropeptides in 16-week old male Snord116^−/−^ mice on chow diet. Quantification of mRNA expression of (**A**) neuropeptide Y (NPY) in the arcuate nuclei of the hypothalamus (ARC); (**B**) proopiomelanocortin (POMC) in ARC; (**H**) tyrosine hydroxylase (TH) in PVN; (**I**) oxytocin (OXY) in the paraventricular nuclei (PVN); (**J**) OXY in the supra optic nuclei (SON); (**K**) orexin (ORX) in the lateral hypothalamic area (LHA); (**L**) melanin concentrating hormone (MCH) in LHA; (**M**) gonadotropin-releasing hormone (GnRH) in the preoptic area (POA); and **N**) growth hormone-releasing hormone (GHRH) in ARC. (**D**) Altered Y1 receptor expression in the hypothalamus using real time PCR. (**C**) Number of alpha-melanocyte-stimulating hormone (α-MSH) positive neurons in the Arc, (**E**) cfos positive neurons in the Arc in response to fasting; (**F**) Percentage of double labelled neurons positive for α-MSH and cFos; (**G**) Representative microphotograph showing the stained neurons of α-MSH and cFos. (White arrows α-MSH neurons, green arrows cFos only stained cells, and yellow arrows indicate double stained neurons; 3V: the third ventricle). Serum concentrations of (**O**) insulin-like growth factor 1 (IGF-1); (**P**) total ghrelin. (*P < 0.05, **P < 0.01, ***P < 0.001, and ****P < 0.0001).

In order to confirm and further evaluate the alterations noted in the gene array experiments, we also performed *in situ* hybridisation for a set of candidate genes known to be critical in hypothalamic energy homeostasis control. In particular the expression of feeding-related neuropeptides, like NPY and POMC in the Arc were determined. Interestingly, as determined by t-test expression levels of both neurotransmitters were significantly increased in Snord116^−/−^ brains (Fig. 4A,B). It is known that NPY and POMC play opposite roles in the regulation of energy homeostasis and the neurons expressing NPY send inhibitory information to the neighbouring POMC cells in the Arc. To understand the potential mechanisms causing the increased expression of both, we firstly confirmed the increased mRNA expression of POMC by counting the number of neurons stained with alpha-melanocyte-stimulating hormone (α-MSH). As shown in the graph (Fig. 4C), a significant increase in the number of α-MSH-labelled neurons was observed in Snord116^−/−^ mice (P < 0.0001) confirming the *in situ* results of POMC mRNA expression also on the protein level. We then tested the relative expression of Y1 receptors in the hypothalamus, as Y1 receptors are known to be expressed on POMC neurons and control their activity. Importantly, a significant decrease of Y1 receptors in the hypothalamus was seen in Snord116^−/−^ mice (P < 0.05) (Fig. 4D), which indicates the connection of the NPY neurons to the POMC cells might be disrupted. To further examine the function of the POMC neurons in the Arc, another group of control and Snord116^−/−^ mice were fasted for 24 hours and the response of Arc neurons to this treatment was analysed by way of counting cfos positive neurons. Fasting induced a significantly higher number of cfos positive neurons in Snord116^−/−^ mice compared to control mice (P < 0.01) (Fig. 4E). Interestingly, when analysing specifically cfos activation of POMC neurons as percentage by using double labelling with an antibody to α-MSH, no significant difference between Snord116^−/−^ and the control mice was observed (Fig. 4F,G). Also of interest is the fact that contrary to previous results no differences in the expression of tyrosine hydroxylase (TH) mRNA and oxytocin was observed in the paraventricular nucleus of the hypothalamus (PVN) (Fig. 4H,I), as well as oxytocin in the supra optic nucleus (Fig. 4J). Interestingly, there was also a small but significant increase (P < 0.05) in the mRNA expression of two orexigenic peptides, orexin (Fig. 4K) and melanin-concentrating hormone (MCH) (Fig.4L) in the lateral hypothalamus. Consistent with the normal reproductive phenotype of Snord116^−/−^ mice, no significant change in the expression of gonadotropin-releasing hormone (GnRH) mRNA in the preoptic area (Fig. 4M) was observed.

Importantly, however, we found a two-fold increase in growth hormone releasing hormone (GHRH) mRNA expression in the Arc in Snord116^−/−^ mice (P < 0.0001) (Fig. 4N) suggesting a compensatory response being induced to counter the reduced growth hormone (GH) action, which is consistent with the observed reduction in serum insulin-like growth factor 1 (IGF-1) levels (P < 0.01) (Fig. 4O) and the reduced stature of Snord116^−/−^ mice. Interestingly, no significant difference in serum ghrelin levels was observed in our Snord116^−/−^ mice (Fig. 4P), which is different to previous reports and suggests that ghrelin does not contribute significantly to the altered feeding phenotype seen in this model. The representative photo micrographs of expression of these neuropeptides in the hypothalamus are shown in Supplementary Figure 3.

### Snord116 deficient mice are resistant to HFD-induced obesity

**Figure 5:**
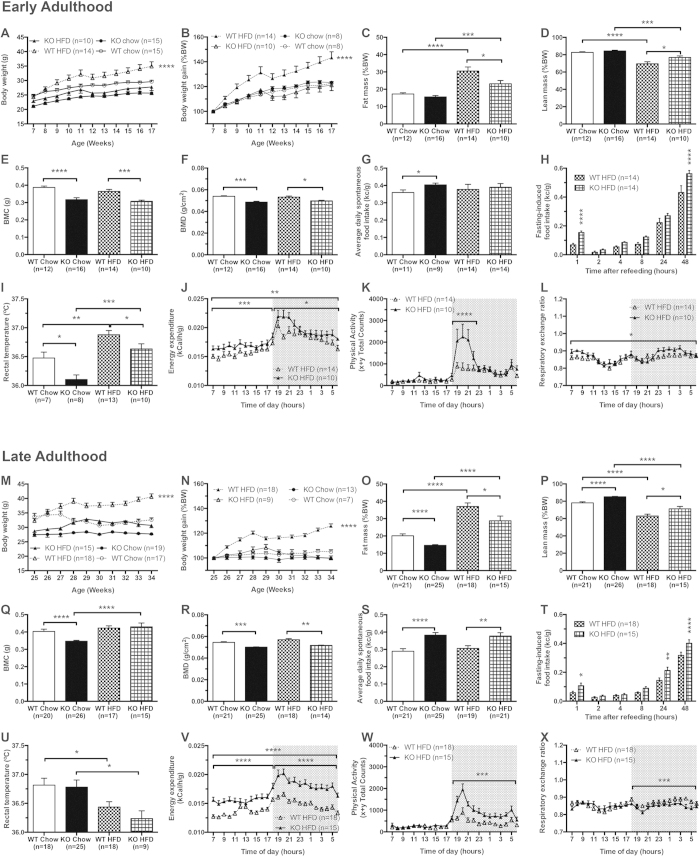
Resistance to high fat diet (HFD) induced obesity in male Snord116^−/−^ mice (—▲—; checked bars) vs. control mice (···△···; squared bars); comparison with male Snord116^−/−^ mice (—●—; black bars) vs. control mice (···O···; white bars) on chow diet. (**A**) Absolute weekly body weight from 7 to 17 weeks of age; (**B**) growth rate normalised to body weight (BW) at 7 weeks of age. In early adulthood (12–16 weeks of age): (**C**) fat mass as a percentage of BW (%BW); (**D**) lean mass (%BW); (**E**) bone mineral content (BMC); (**F**) bone mineral density (BMD); (**G**) spontaneous 24-hour calorie intake as a percentage of body weight (%BW), expressed as the average of triplicate readings over three consecutive days; (**H**) fasting-induced calorie intake (%BW); (**I**) core body temperature; (**J**) 24-hour energy expenditure; (**K**) 24-hour physical activity; (**L**) 24-hour respiratory exchange ratio (RER). (**M**) Absolute weekly body weight from 25 to 34 weeks of age; (**N**) growth rate normalised to BW at 25 weeks of age. In late adulthood (28-32 weeks of age): (**O**) fat mass (%BW); (**P**) lean mass (%BW); (**Q**) BMC; (**R**) BMD; (**S**) spontaneous 24-hour calorie intake (%BW), expressed as the average of triplicate readings over three consecutive days; (**T**) fasting-induced calorie intake (%BW); (**U**) core body temperature; (**V**) 24-hour energy expenditure; (**W**) 24-hour physical activity; (**X**) 24-hour RER. (*P < 0.05, **P < 0.01, ***P < 0.001, and ****P < 0.0001).

Given that the typical delayed-onset obesity seen in PWS subjects was not observed in Snord116^−/−^ mice when fed a normal chow diet, we next investigated whether obesity could be induced by HFD. To this end, Snord116^−/−^ and control mice were fed a HFD from the age of 6 weeks for the early adult study and from the age of 25 weeks for the late adult study until the end of the monitoring period. As shown in Fig. 5A,B and analysed by two-way ANOVA, in early adulthood the body weight of control mice on HFD increased approximately 44% from baseline over the 11 week monitoring period whereas Snord116^−/−^ mice only showed an increase of 21% (P < 0.0001, F = 8.186), a similar figure to the growth rate observed in these mice when fed a chow diet. Importantly, however, despite the lack of significant body weight gain compared to the chow condition, fat mass analysed by one-way ANOVA was significantly increased in the Snord116^−/−^ mice on HFD (P < 0.001, F = 20.37) (Fig. 5C,D). A similar phenotype was also observed for mice in the late adult stage where overall body weight increase was not significantly different between Snord116^−/−^ mice on chow or HFD, but fat mass was elevated on HFD (Fig. 5M–P) suggesting a contribution of the Snord116 gene products in controlling fat mass.

### Increased Energy Intake and Expenditure of Snord116^−/−^ mice on HFD

To further investigate the influence of HFD on general aspects of energy homeostasis, we monitored food intake and energy expenditure in Snord116^−/−^ and control mice. Within each genotype, caloric intake was not significantly different between mice on chow or HFD when compared as a percentage of body weight. However, in early adulthood control mice on HFD had greater caloric intake than their counterparts on chow, whereas the Snord116^−/−^ mice remained the same on HFD as they did on chow diet (Fig. 5G). Importantly, in late adulthood, Snord116^−/−^ mice continued to exhibit hyperphagia, with food intake significantly higher than that seen in control mice on the same diet (P < 0.01, F = 8.727) (Fig. 5S). Moreover, fasting-induced food intake in Snord116^−/−^ mice analysed by two-way ANOVA followed by Bonferroni post-tests was also significantly increased in both early and late adulthood (Fig. 5H,T).

HFD also led to increased energy expenditure and physical activity in Snord116^−/−^ mice, thereby somewhat offsetting the increase in food intake (Fig. 5J,K,V,W). Consistent with being on HFD, both genotypes showed a decrease in overall RER compared to their respective chow diet controls (Figs 5L,X and 3F,L). In addition, RER of Snord116^−/−^ mice on HFD increased in early adulthood (P < 0.05), but reduced in the dark phase during late adulthood (P < 0.001) (Fig. 5L,X).

### Selective deletion of Snord116 only in NPY neurons replicates the global deletion phenotype

The data obtained from microarray and *in situ* hybridization analysis in Snord116^−/−^ mice revealed the alteration of the mRNA expression in both NPY and POMC neurons in the hypothalamus, the primary appetite regulating neurons that control feeding behaviour. Interestingly however, the normally observed counter-regulatory up- and down-regulation of NPY and POMC mRNA, respectively, under negative energy balance condition was not observed in this model. This suggests that the dominant role of the inhibiting projection from NPY neurons to POMC neurons does not function in the normal way and that Snord116 may be critical for this process to occur.

**Figure 6:**
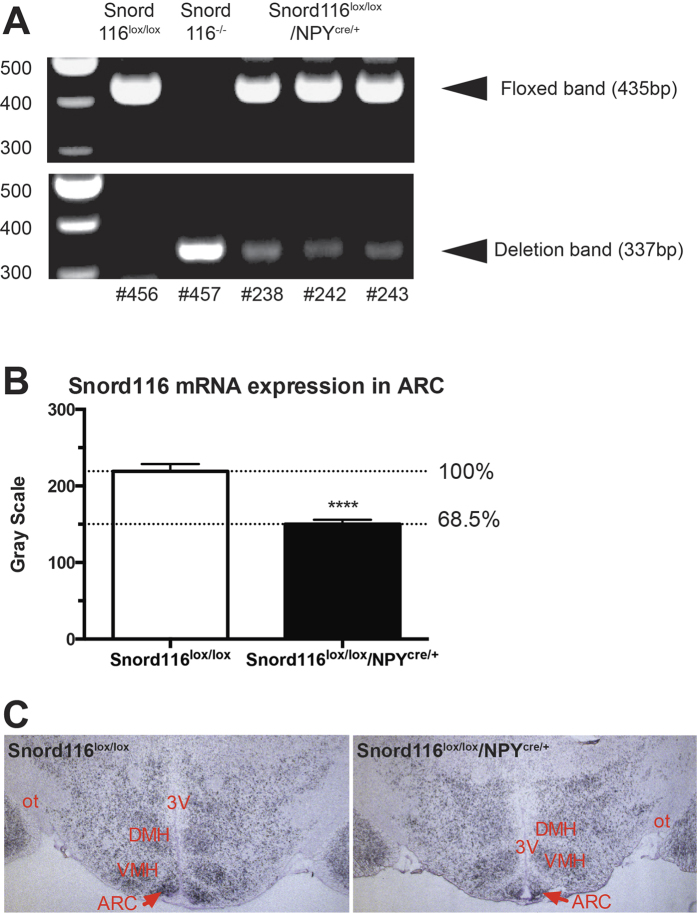
Generation of Snord116lox/lox/NPYcre/ + mice. (**A**) PCR genotyping results of Snord116lox/lox/NPYcre/+ mice. (**B**) Snord116 mRNA expression in the arcuate nuclei of the hypothalamus (ARC); **C**) Photographs of the expression of Snord116 mRNA in ARC detected by *in situ* hybridisation (left panel: Snord116^lox/ox^, right panels: Snord116^lox/lox^/NPY^cre/+^).

In order to test this hypothesis, a mouse line with specific deletion of Snord116 only in NPY neurons was generated. Briefly, floxed Snord116 mice (Snord116^lox/lox^) were crossed with a transgenic line that drives Cre gene expression under the control of the NPY promoter (NPY^cre/+^) to generate double heterozygous mice (Snord116^lox/+^/NPY^cre/+^). These mice were then crossed again with Snord116^lox/lox^ mice to generate Snord116^lox/lox^/NPY^cre/+^ mice and controls. The successful deletion of the Snord116 gene cluster was then confirmed by PCR on DNA isolated from the hypothalamus of these mice (Fig. 6A) as well as by *in situ* hybridisation of brain sections from Snord116^lox/lox^/NPY^cre/+^ mice compared to WT controls (P < 0.0001, analysed by t-test) (Fig. 6B,C).

**Figure 7:**
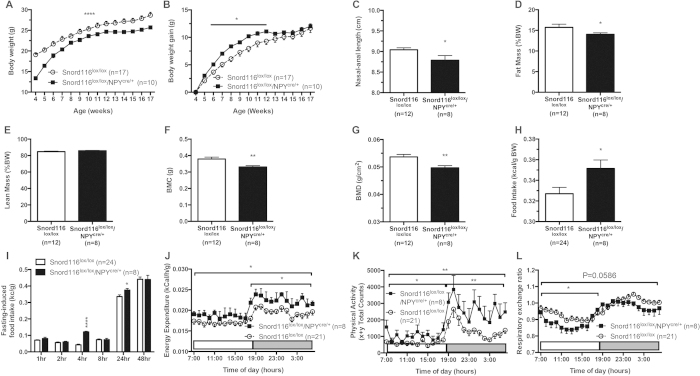
Selective deletion of Snord116 only in NPY neurons replicates the global deletion phenotype. (**A**) Absolute weekly body weight (BW) from 4 to 17 weeks of age; (**B**) growth rate normalised to BW at 4 weeks of age; (**C**) nasal-anal length; (**D**) fat mass as a percentage of BW (%BW); (**E**) lean mass (%BW); (**F**) bone mineral content (BMC); (**G**) bone mineral density (BMD); (**H**) spontaneous 24-hour calorie intake as a percentage of body weight (%BW), expressed as the average of triplicate readings over three consecutive days; (**I**) fasting-induced calorie intake (%BW); **J**) 24-hour energy expenditure; (**K**) 24-hour physical activity; (**L**) 24-hour respiratory exchange ratio (RER). (*P < 0.05, **P < 0.01, ***P < 0.001, and ****P < 0.0001).

Snord116^lox/lox^/NPY^cre/+^ and control mice were then investigated by the same multi-measure metabolic profiling procedure that was used for the globally deleted mice. As shown in Fig. 7A, similar to the Snord116^−/−^ mice, Snord116^lox/lox^/NPY^cre/+^ mice displayed low birth weight compared to controls and this reduced weight persisted until adulthood (P < 0.0001 and F = 6.642, analysed by two-way ANOVA). More importantly, however, body weight gain of Snord116^lox/lox^/NPY^cre/+^ mice over this period was actually increased (P < 0.05) (Fig. 7B), again almost identical to the phenotype seen in the global deleted mice. This reduced body weight was also reflected in a shorter nasal-anal length (P < 0.05 by t-test) (Fig. 7C) and was associated with a significantly reduced fat mass (P < 0.05) but did not alter lean mass when determined by DXA (Fig. 7D,E). These DXA data are confirmed by significant alterations in weights of dissected WAT depots from different regions (data not shown). Collectively, these data demonstrate that selective deletion of the Snord116 gene cluster only from NPY producing neurons replicates the general phenotype of the globally deleted Snord116^−/−^ mice and suggest a critical role of Snord116 in NPY neurons.

To further investigate the influence of lack of Snord116 expression in NPY neurons on general aspects of energy homeostasis, we monitored food intake and energy expenditure in Snord116^lox/lox^/NPY^cre/+^ and control mice. Importantly, despite the reduced body weight of Snord116^lox/lox^/NPY^cre/+^ mice, a significant increase in spontaneous food intake was observed in these mice (P < 0.05, t-test) (Fig. 7H). On the other hand, fasting-induced food intake showed no significant difference between Snord116^lox/lox^/NPY^cre/+^ mice and the controls with the exception of the increase at the 4-hour and 24-hour time points (Fig. 7I). When investigating energy expenditure (P < 0.05) and physical activity (P < 0.01) in Snord116^lox/lox^/NPY^cre/+^ mice a clear increase could be observed (Fig. 7J,K). This increase in energy expenditure might also be one reason why the increase in food intake does not result in a higher body weight in these mice. Furthermore, Snord116^lox/lox^/NPY^cre/+^ mice showed a decrease in overall RER compared to their controls (Fig. 7L) indicating a shift towards greater use of fatty acids as their fuel source.

**Figure 8:**
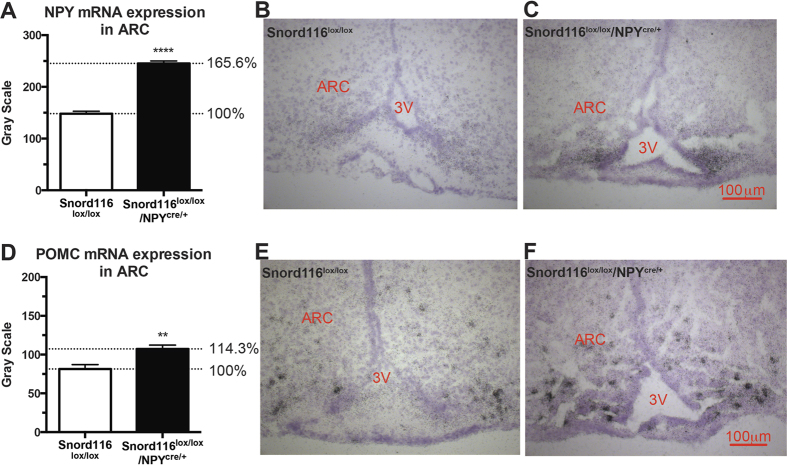
Increased expression of NPY and POMC in the arcuate nuclei (ARC) in 16-week old male Snord116^lox/lox^/NPY^cre/+^ mice, detected by *in situ* hybridisation. Quantification of mRNA expression of neuropeptide Y (NPY) (**A**) and proopiomelanocortin (POMC) (**D**) in the ARC with representative microphotographs followed respectively (**B,C,E,F**).

In order to confirm and further investigate the direct influence of Snord116 on NPY neurons we performed *in situ* hybridisation. Again, identical to the global deletion model, both NPY and POMC mRNA expression in the Arc were significantly upregulated (P < 0.0001 for NPY expression and P < 0.01 for POMC) (Fig. 8A,D).

Taken together, all the observed phenotypes related to energy homeostasis and metabolism in the Snord116^lox/lox^/NPY^cre/+^ mice are almost the same as the results seen in global deleted Snord116^−/−^ mice, indicating the critical role of Snord116 in NPY neuronal function.

## Discussion

This study provides clear evidence that the lack of the Snord116 gene cluster in mice contributes to the development of a subset of PWS phenotypes seen in humans, including low postnatal body weight, short stature, reduced bone mass, hyperphagia and altered energy expenditure. However, the second phase of classical PWS development – the establishment of an obese phenotype in later life – is absent and Snord116^−/−^ mice are actually resistant to HFD-induced obesity. Interestingly, the absence of Snord116 expression during embryonic development leads to significant alterations of hypothalamic neuropeptide expression patterns, which are consistent with the observed increases in feeding behaviour and energy expenditure particular in the early development phase. Importantly, restricting the deletion of the Snord116 cluster to only one type of neurons, NPY neurons (known for their important role in feeding behaviour and energy homeostasis regulation), an almost identical phenotype to the global deleted model was observed. This suggests a critical role for Snord116 function in this particular set of neurons.

Our Snord116 knockout model differs from previously published ones^21^,^30^, in that we deleted both alleles from the germline. Most genomic imprinting studies in PWS assume a simple pattern of imprinting, where the paternally inherited copy is expressed while the maternal copy is silenced^27^. However, quantitative studies on the effects of imprinted alleles have also shown that more complex patterns of expression can exist, which are dependent on the genetic information derived from both parents^28^. Therefore to investigate the full phenotype of lack of Snord116 gene expression, instead of using heterozygous deletion mice, a complete Snord116 germline deletion model was used in this study.

While there is overlap with the results obtained from the metabolic analysis of the heterozygous model, there are also several features that are different. For example, our gene profiling experiments in the hypothalamus comparing Snord116^−/−^ and control mice at the stage of early adulthood revealed a clear pattern of alterations, particularly in genes encoding known regulators of energy intake and energy homeostasis (as identified by gene pathway analysis), which were not found in the earlier studies. These changes were also confirmed by *in situ* hybridisation experiments, which showed significant up-regulation of neuropeptide genes such as GHRH, NPY, POMC, orexin and MCH. Elevated ghrelin levels were identified as one of the major contributors to the hyperphagia phenotype in a previous study^21^, a result which could not be confirmed in our model, where serum ghrelin levels between Snord116^−/−^ and control mice were not significantly different. In line with one human PWS study reporting that ghrelin concentrations change during development^31^, the difference between previous reports and our result could be due to the selection of different time intervals for measurement. Importantly, GHRH mRNA levels showed a strong elevation, which is consistent with the strong trend to reduced serum IGF-1 levels and another indicator of reduced body weight and lean phenotype. Changes in these parameters are also seen in humans with PWS where the GH axis is affected showing low peak GH responses to provocative stimuli and reduced spontaneous GH secretion and low serum IGF-1 levels^32^. Clinical characteristics ascribable to GH deficiency, namely short stature and reduced bone mineral density (BMD), are also paralleled in mice lacking Snord116.

Interestingly and consistent with the previous model is the fact that the lack of Snord116 expression does not lead, like in humans, to increased body weight and an obese phenotype, even under conditions of a high caloric HFD, although fat mass does increase under these conditions. The most likely explanation for this discrepancy might be the concomitant increase in energy expenditure that compensates for most of the additional energy intake under both chow and HFD conditions. The increased activity, particularly in the dark phase, might be an additional contributor to the elevated energy expenditure. Interestingly, the preferred nutrient source for the Snord116^−/−^ mice does also change from a carbohydrate preference under chow conditions to a preference for fat under HFD conditions. Of note, it can not be completely excluded that due to the smaller body size of the Snord116^−/−^ mice they might lose more heat and require additional energy to compensate for this by increasing their food intake. Further studies under thermo-neutral conditions will be required to determine this possibility.

The energy homeostasis phenotype in Snord116^−/−^ mice is markedly different to that of humans with PWS, possibly explaining the discrepancy in the incidence of obesity between the two models. Individuals with PWS have lower physical activity than healthy controls^33^,^34^, and have been found to have reduced energy expenditure^33^,^35^,^36^,^37^, both of which are consistent with, and may stem from, the low lean mass commonly observed in PWS.

The observation that particular neurotransmitters within the hypothalamus that are known to be critical components in the control of feeding and energy homeostasis are significantly altered in their expression when Snord116 is deleted suggests a functional link between this snoRNA and appetite control. Intriguingly however, the changes seen in NPY and POMC mRNA, which are both upregulated under this conditions, are surprising since they normally show opposing expression levels in response to situations of positive or negative energy balance. It has also now been clearly established that NPY has the dominant role in this counter regulatory process and high NPY levels would normally cause the down-regulation of POMC mRNA. The fact that this is not the case in this model suggests a vital connection or functional response, most likely through alteration in Y1 receptor signalling, is induced as a consequence of lack of Snord116 expression. This is also confirmed by our results with the conditional model where the selective deletion of Snord116 only in NPY neurons is sufficient to replicate the global deletion effect on energy homeostasis regulation and importantly also reproduces the same dys-regulation of NPY and POMC mRNA expression in the hypothalamus.

The general pattern of alterations of these neuropeptides fits with a notion of increased feeding behaviour with the exception of the elevated POMC mRNA levels, which normally would be associated with an anorexic effect. POMC neurons are strongly influenced by signalling from NPY neurons that suppresses POMC neuronal activity. The presence of high NPY and high POMC levels might indicate that this fundamental control mechanism of NPY neurons over POMC neurons to balance out energy homeostasis is disrupted and that in the early stages of development the anorexic function of the POMC neurons dominates with the increased drive by NPY for feeding taking hold later in life consistent with the significant increased food intake at this stage of life of the Snord116^−/−^ mice.

The mechanism behind this control of NPY neuronal function is not entirely clear. Snord116 has been classified as member of the C/D box snoRNAs that are implicated in the modification of other RNA molecules and also in the process of alternative splicing. Our analysis of altered splicing of hypothalamic transcripts from Snord116^−/−^ compared to control mice did not find any significant differences suggesting that this is not the major function of Snord116 or that the changes are very subtle and not clearly identifiable by this methodology. It is also important to keep in mind that Snord116 is being produced as a cleavage product out of intronic sequences of the large SNURF-SNRPN transcripts (Fig. 1A) and the deletion of the Snord116 cluster removes approximately 150 kb of genomic sequence, including some of the exons of the SNURF-SNRPN transcripts. While there was no obvious difference in the expression levels of these large non-coding RNA transcripts in Snord116^−/−^ mice seen from the array data, it is possible that the overall structure of these RNA molecules was altered due to the missing sequences contained in the Snord116 cluster and through that contributed to an altered function of NPY neurons.

Taken together, the analysis of germline homozygous Snord116 knockout mice has confirmed a critical role of this snoRNA in controlling brain development and the establishment of neuronal pathways that are important to control feeding behaviour and maintain energy homeostasis. Furthermore, the fact that the exact phenotype of this global deletion model can be replicated in a NPY neuron specific Snord116 knockout model highlights the importance of this population of neurons and their critical role in the development of a hyperphagic phenotype. While the overall observed phenotype seen in these mouse models does not completely replicate the dramatic changes seen in humans with PWS, there are several striking similarities confirming Snord116 contributes to the aetiology of PWS.

## Materials and Methods

### Animals

Research procedures and animal care for this study were approved by Garvan Institute/St. Vincent’s Hospital Animal Ethics Committee and were in agreement with the Australian Code of Practice for the Care and Use of Animals for Scientific Purpose. Mice were housed under conditions of controlled temperature (22 ± 1 °C as room temperature and 30 ± 1 °C for thermo-neutrality) and illumination (12-hour light-dark cycle, with lights on at 07:00 am and off at 07:00 pm). Animals had *ad libitum* access to either a normal chow diet (8% calories from fat, 21% calories from protein, 71% calories from carbohydrate, and 2.6 kcal/g; Gordon’s Specialty Stock Feeds, Yanderra, NSW, Australia) or HFD (46% calories from fat, 21% calories from protein, 33% calories from carbohydrate, 4.72 kcal/g). Water was unrestrictedly available. Where applicable, HFD replaced the normal chow diet six weeks before commencement of metabolic monitoring and was maintained until study completion. Body weight was measured weekly from the beginning of HFD treatment for high fat fed groups and from the beginning of the metabolic monitoring period for normal chow fed groups.

### Core temperature

As the measurement of body temperature in mice is highly sensitive, measuring procedures were kept as uniform as possible. Mice were restrained and the rectal probe was inserted at 9:00 am for three consecutive days. The average of triplicate readings was calculated as basal body temperature.

### Feeding and energy homeostasis studies

To investigate energy intake, both 24-hour spontaneous food intake and fasting-induced food intake were measured. Mice were individually housed and fed powdered diet for 3 days in preparation for feeding studies. They were then transferred into Nalgene metabolic cages (Medtex, Notting Hill, VIC), which are specially designed for monitoring food intake, water consumption, faeces and urine production, and acclimatised for 24 hours. Spontaneous 24-hour food intake was monitored over three consecutive days and the average of triplicate readings was taken as basal food intake. For fasting-induced food intake, food consumption was monitored at the time points of 1, 2, 4, 8, 24 and 48 hours after an 18-hour fast. Body weight was recorded daily during the measurement of spontaneous food intake and at the time points of 0, 8, 24 and 48 hours for fasting-induced food intake. Food intake was calculated as calorie intake and normalised to body weight of individual mice.

Energy expenditure was assessed using an eight-chamber open-circuit Oxymax system (Oxymax Series; Columbus Instruments, Columbus, OH, USA). Mice were acclimatised in the chambers for 24 hours, followed by a 24-hour continuous measurement of oxygen consumption and carbon dioxide production. RER and heat production were calculated from these parameters. Energy expenditure was determined by correcting heat production for total lean and fat mass. Physical activity was continuously recorded using a passive infrared detector (OPTO-M3 sensor). Physical activity was expressed as the hourly sum of total X and Y plane beam breaks over 24 consecutive hours; calorimetry data were expressed as hourly means over 24 consecutive hours.

### Dual energy X-ray absorptiometry

DXA was used to determine body composition. Mice anaesthetised with isoflurane were scanned using a Lunar PIXImus2 mouse densitometer (GE healthcare, Waukesha, WI, USA). Whole body lean mass, fat mass, BMD and bone mineral content (BMC) were determined for individual mice.

### Intraperitoneal insulin tolerance test and glucose tolerance test

Glucose metabolism was investigated by insulin and glucose tolerance tests. For ITT, mice that had been fasted for five hours were given an intraperitoneal injection of insulin (0.75 IU/kg) (Novo Nordisk Pharmaceuticals, Baulkham Hills, NSW, Australia). For GTT, a dose of a 10% D-glucose solution (1.0 g/kg) (Astra Zeneca, North Ryde, NSW, Australia) was injected intraperitoneally after an overnight fast. Blood glucose levels were measured in tail blood using an Accu-chek® Go glucometer (Roche, Dee Why, NSW, Australia) immediately before injection and at 15, 30, 60 and 90 minutes post-injection.

### Tissue collection

Mice were culled by cervical dislocation followed by decapitation. Trunk blood was collected, from which serum was obtained after centrifugation at 13 000 rpm for 4 minutes and stored at −20 °C for further analysis. Fresh brains were collected and frozen flat on dry ice, then stored at −80 °C. The interscapular brown adipose tissue (BAT) and WAT from the inguinal, epididymal, retroperitoneal and mesenteric deposits were excised and weighed. The organs, including gonads, spleen, pancreas, kidney, liver and heart, were also excised and weighed. Tissue and organ weight was expressed as a percentage of body weight.

### *In situ* hybridisation and densitometry

Fresh frozen brains were sectioned at 30 μm thickness and thaw-mounted on Superfrost Plus® glass microscope slides (Lomb Scientific Pty Ltd., NSW 2229, Australia). *In situ* hybridisation was performed, as previously described^38^. Briefly, matching hypothalamic sections of deletion and control mice were hybridised with candidate mRNAs, which were labelled with [^35^S] thio-dATP (Amersham Pharmacia Biotech, Buckinghamshire, UK) using terminal deoxynucleotidyltransferase (Roche, Mannheim, Germany). Silver grain densities of labelled mRNAs were analysed and compared using ImageJ software (US National Institutes of Health).

DNA oligonucleotides used included those complementary to the mRNAs of mouse Snord116, mouse GHRH, mouse NPY, mouse POMC, mouse MCH, mouse orexin, mouse GnRH, mouse oxytocin and mouse TH (see Supplementary Table 2).

### Quantitative real-time PCR

To test the expression of Y1 receptors in the hypothalamus, total RNA from the hypothalamic region of both Snord116^−/−^ mice and controls was isolated using Trizol^®^ Reagent (Sigma, St Louis, MO, USA). Quantitative real-time PCR was carried out following the protocol as previously described^39^. Briefly, the total RNA was reverse transcribed into cDNA by the Superscript III First-strand Synthesis System (Invitrogen, Mount Waverley, VIC, Australia). Expression of Y1-receptor gene and the housekeeping gene-ribosomal protein L13A (RPL-13A), was quantified on a LightCycler^®^ (LightCycler^®^ 480, Roche Applied Science, Germany) using SensiMix™ Probe (Bioline Australia Pty Ltd, Alexandria, NSW, Australia). The latter was used to normalize the relative expression level of Y1 receptors.

### Immunohistochemistry

Mice were anaesthetized and perfused with 0.9% saline and 4% paraformaldehyde (PFA), followed by post-fixative in 4% PFA for 2 hours and 30% sucrose overnight. Brains were then sectioned at 40 μm interval and stored in cryoprotectant at −20 °C until use. The brain regions of interest were identified according to the maps in the Mouse Brain in Stereotaxic Coordinates^40^ and the immunohistochemistry were implemented following the protocol described in Supporting Information Experimental Procedures.

In order to further verify the up-regulated mRNA expression of POMC, the identified sections at 120-μm intervals through the Arc for each mouse (n = 6 for each group) were selected and the expression of α-MSH, one of the downstream protein products of POMC, was tested using sheep anti-mouse α-MSH antiserum (1:1000; AB5087, Chemicon, Temecula, CA, USA). The numbers of α-MSH stained cells were then counted by single-blind method and microscope images were digitally captured with the attached camera. Data were presented as the average number of α-MSH stained neurons per section in each nucleus.

To examine the neural activity of the POMC cells with fasting, as indicated by cFos staining, double staining immunohistochemistry of α-MSH and cFos (rabbit anti-mouse cFos, 1:1000; Santa Cruz Biotechnology Inc., Santa Cruz, CA, USA) was performed in the Arc sections of Snord116^−/−^ mice and controls after those animals were fasted for 24 hours. The single and double cFos-labelled α-MSH neurons were counted using fluorescent microscope (Leica Microsystems Pty Ltd, North Ryde, Australia). Data were represented as percentage of double labelled cells in α-MSH immunoreactive neurons per section in the Arc and pictures were taken with the attached camera.

### Microarray and bioinformatics analysis

RNA was extracted from hypothalamic tissue of wildtype and Snord116^−/−^ mice using TRIzol® reagent (Sigma) according to the manufacturer’s instructions. RNA samples were subsequently checked for consistent quality and quantified using the Agilent 2100 Bioanalyser (Agilent Technologies) according to the manufacturer’s instructions. Labelling, hybridisation to Affymetrix Mouse Gene 1.0 ST Array was performed by the Ramaciotti Centre for Gene Function Analysis (University of New South Wales, Sydney, Australia). 6 arrays were performed on 3 separate wildtype samples and 3 separate Snord116^−/−^ samples.

Normalisation and probe set summarisation were performed using the robust multi- chip average^41^ implemented in the Affy library^42^ from R/Bioconductor^43^. Control probe sets were removed leaving 28853 probe sets on the array. Differential gene expression was assessed for each probe set using Limma^44^. Gene Set Enrichment Analysis^45^ was run with the GenePattern tool GSEApreranked using a ranked list of t-statistics derived from Limma analysis against c2_all, c3_all and c5_bp of the Molecular Signatures Database (MSigDB)^45^. These analyses were performed using Gene Pattern software^46^ and are available at http://pwbc.garvan. unsw.edu.au/gp. To analyse for identifying transcripts that are alternative spliced we employed Partek software.

### Biochemical analyses

Serum IGF-1 was measured by enzyme-linked immunosorbent assay (ALPCO Diagnostics, Salem, NH, USA). Serum ghrelin was measured by radioimmunoassay (Millipore Co., Billerica, MA, USA).

### Statistical analyses

All data are presented as means ± SEM. T-tests were used to analyse the difference of body composition, blood glucose level, daily spontaneous food intake and rectal temperature between Snord116^−/−^; Snord116^lox/lox^/NPY^cre/+^ mice and their respective controls. T-test was also employed to analyse the expression data of hypothalamic neuropeptides, the cell counting data and serum hormones levels. One-way ANOVA with Bonferroni post-tests was chosen to compare body compositions, daily spontaneous food intake and rectal temperature between the mutant mice and their controls when they were fed with HFD. Difference of body weight, ITT, GTT, fasting-induced food intake, physical activity and RER between Snord116^−/−^ /Snord116^lox/lox^/NPY^cre/+^ mice and their respective controls with either normal chow diet or HFD was analysed by two-way ANOVA with Bonferroni post-tests to compare each group to the others. ANCOVA (with lean mass as a co-variant) was used to analyse energy expenditure data. Statistical analyses were performed with SPSS for Mac OS X version 16.0.1 (SPSS Inc, Chicago, IL, USA). Statistical significance was defined as P < 0.05.

## Additional Information

**How to cite this article**: Qi, Y. *et al.* Snord116 is critical in the regulation of food intake and body weight. *Sci. Rep.* **6**, 18614; doi: 10.1038/srep18614 (2016).

## Supplementary Material

Supplementary Information
