# Supplementary material for: Snord116 is critical in the regulation of food intake and body weight
Source: Sci Rep. 2016 Jan 4;6:18614. doi: 10.1038/srep18614 (PMC4698587; doi:10.1038/srep18614)
Supplement: Supplementary Information [file srep18614-s1.pdf]

Title: Snord116 is critical in the regulation of food intake and body weight

Author list: Yue Qi[1], Louise Purtell[2], Melissa Fu[1], Nicola J. Lee[1], Julia Aepler[1], Lei Zhang[1], Kim Loh[1], Ronaldo F. Enriquez[3], Paul A. Baldock[3], Sergei Zolotukhin[4], Lesley V. Campbell[2] & Herbert Herzog[1,5]

Supplementary Figure 1

Low body weight, increased growth rate in early stage, altered body composition, and increased energy intake and energy expenditure in female Snord116$^{-/-}$ mice (**—●—;** black bars) vs. control mice (⋯O⋯; white bars) on chow diet.

A) Absolute weekly body weight from 4 to 35 weeks of age; **B**) growth rate normalised to body weight (BW) at 4 weeks of age;

In early adulthood (12-16 weeks of age): **C**) fat mass as a percentage of BW (%BW); **D**) lean mass (%BW); **E**) bone mineral content (BMC); **F**) bone mineral density (BMD); **G**) blood glucose curves during intraperitoneal glucose tolerance test as a percentage of fasting glucose levels (GTT); **H**) blood glucose curves during insulin tolerance test as a percentage of fasting glucose levels (ITT); **I**) spontaneous 24-hour calorie intake (%BW), expressed as the average of triplicate readings over three consecutive days; **J**) fasting-induced calorie intake (%BW); **K**) 24-hour energy expenditure; **L**) 24-hour physical activity; **M**) 24-hour respiratory exchange ratio (RER).

In late adulthood (28-32 weeks of age): **N**) fat mass (%BW); **O**) lean mass (%BW); **P**) BMC; **Q**) BMD; **R**) GTT; **S**) ITT; **T**) spontaneous 24-hour calorie intake (%BW); **U**) fasting-induced calorie intake (%BW); **V**) 24-hour energy expenditure; **W**) 24-hour physical activity; **X**) RER.

(* P<0.05, ** P<0.01, *** P<0.001, and **** P<0.0001)

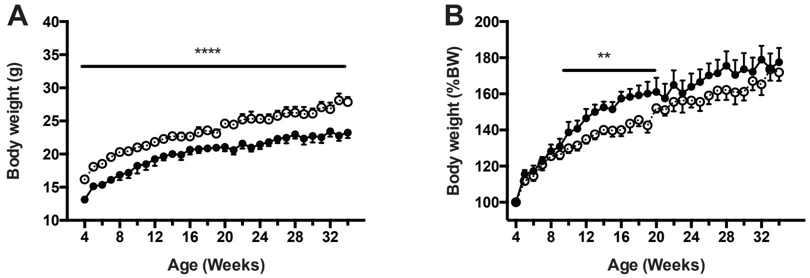

Early adulthood

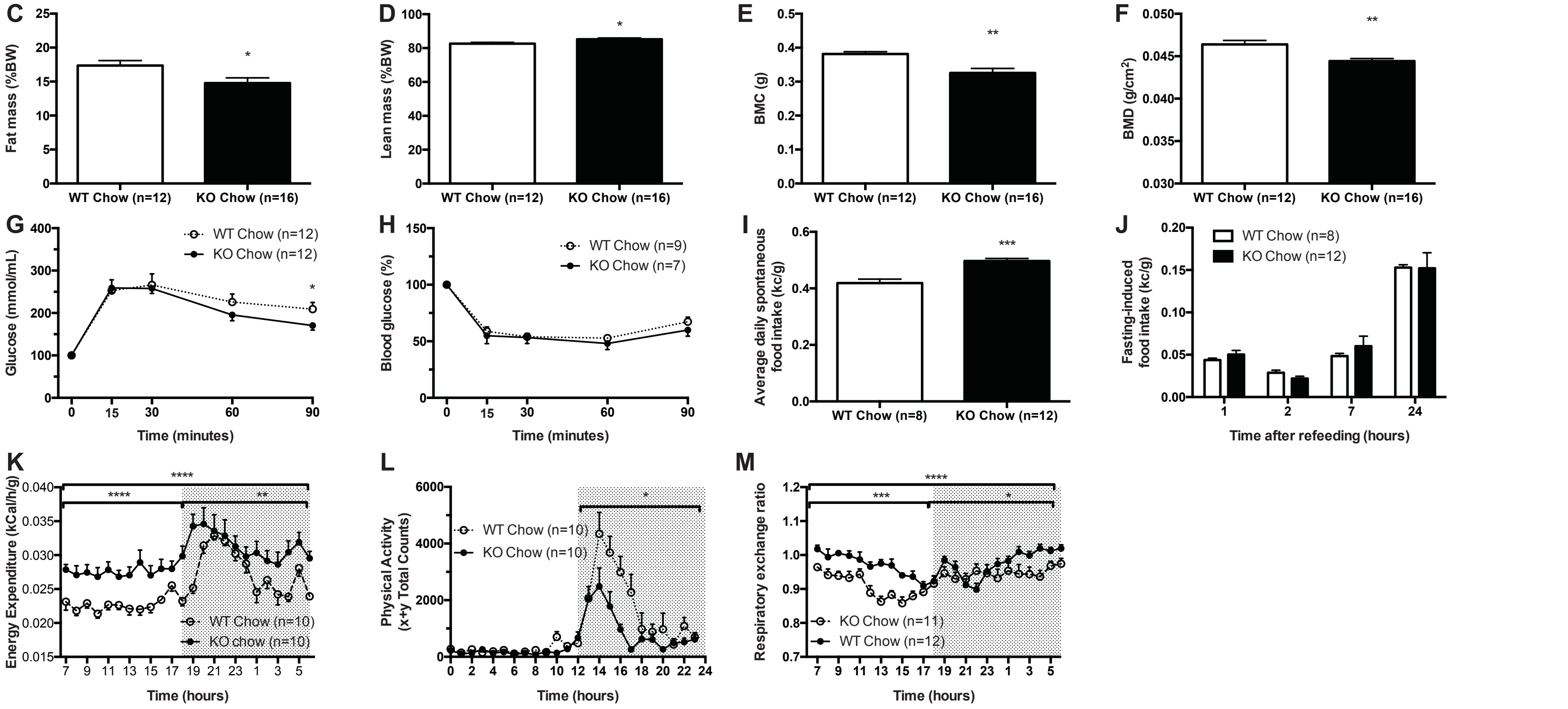

Late adulthood

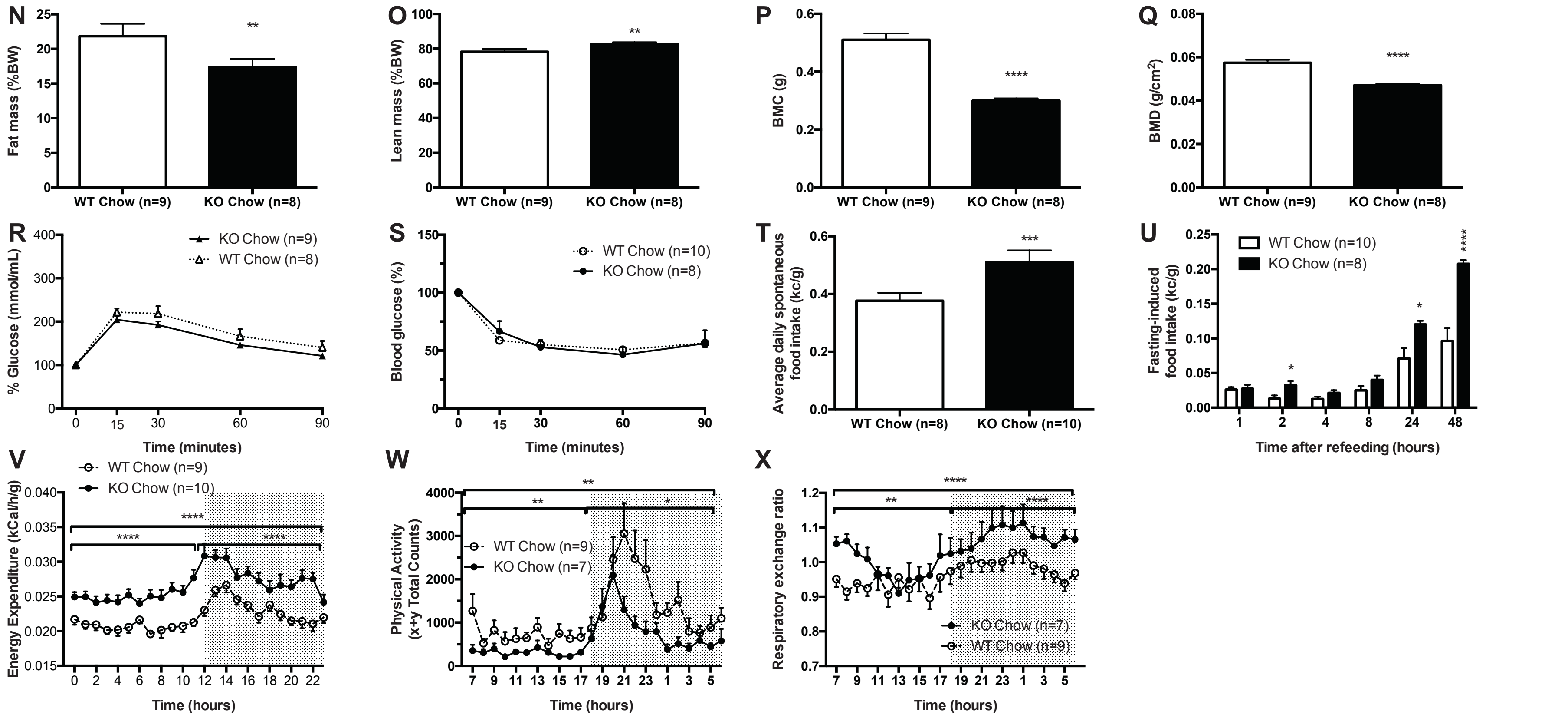

Supplementary Figure 2

Male-and-female combined graphs of absolute weekly body weight **(A)**, body weight gain **(B)** and food intake **(C)** and the statistic comparisons between groups **(D)** showing the significant difference between genotypes (Snord116$^{-/-}$ and the wild type) and genders (male and female).

A

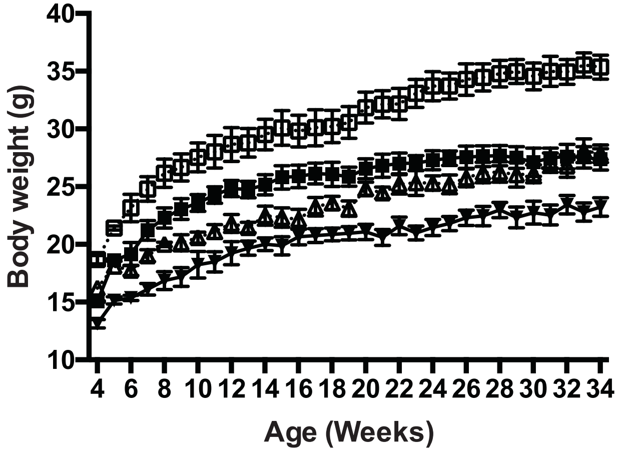

B

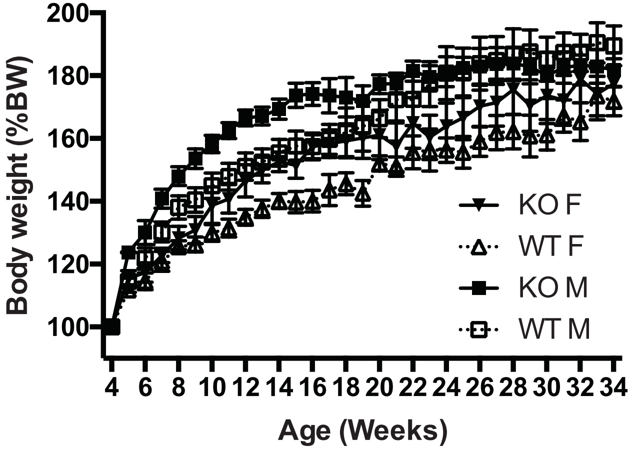

C

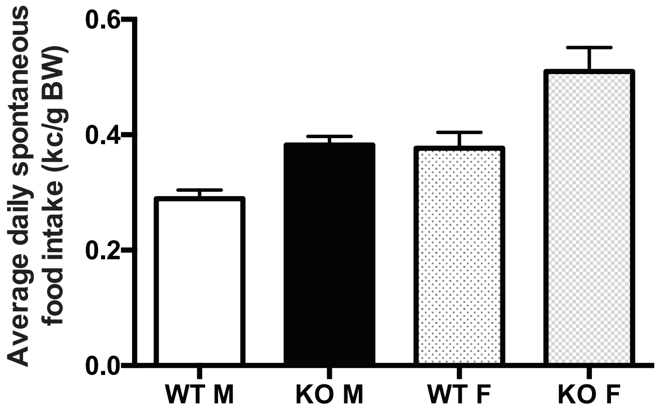

D

Tukey's multiple comparisons test	Body weight significant	Body weight gain (Week 6 to 18; %BW)	Food intake significant
WT M vs. KO M	***	***	***
KO M vs. KO F	**	***	**
WT F vs. KO F	*	**	*
WT M vs. WT F	**	****	ns

Supplementary Figure 3

Expression of appetite-related neuropeptides in hypothalamic nuclei, detected by *in situ* hybridisation (black dots indicated by red arrows; left panels: wildtype (WT), right panels: Snord116$^{-/-}$; neuropeptide Y (NPY), orexin (ORX), melanin concentrating hormone (MCH), proopiomelanocortin (POMC), gonadotropin-releasing hormone (GnRH), growth hormone-releasing hormone (GHRH), third ventricle (3V), arcuate nuclei (ARC), lateral hypothalamic area (LHA), optic chiasm (oc), optic tract (ot), preoptic area (POA), ventromedial nuclei (VMH)).

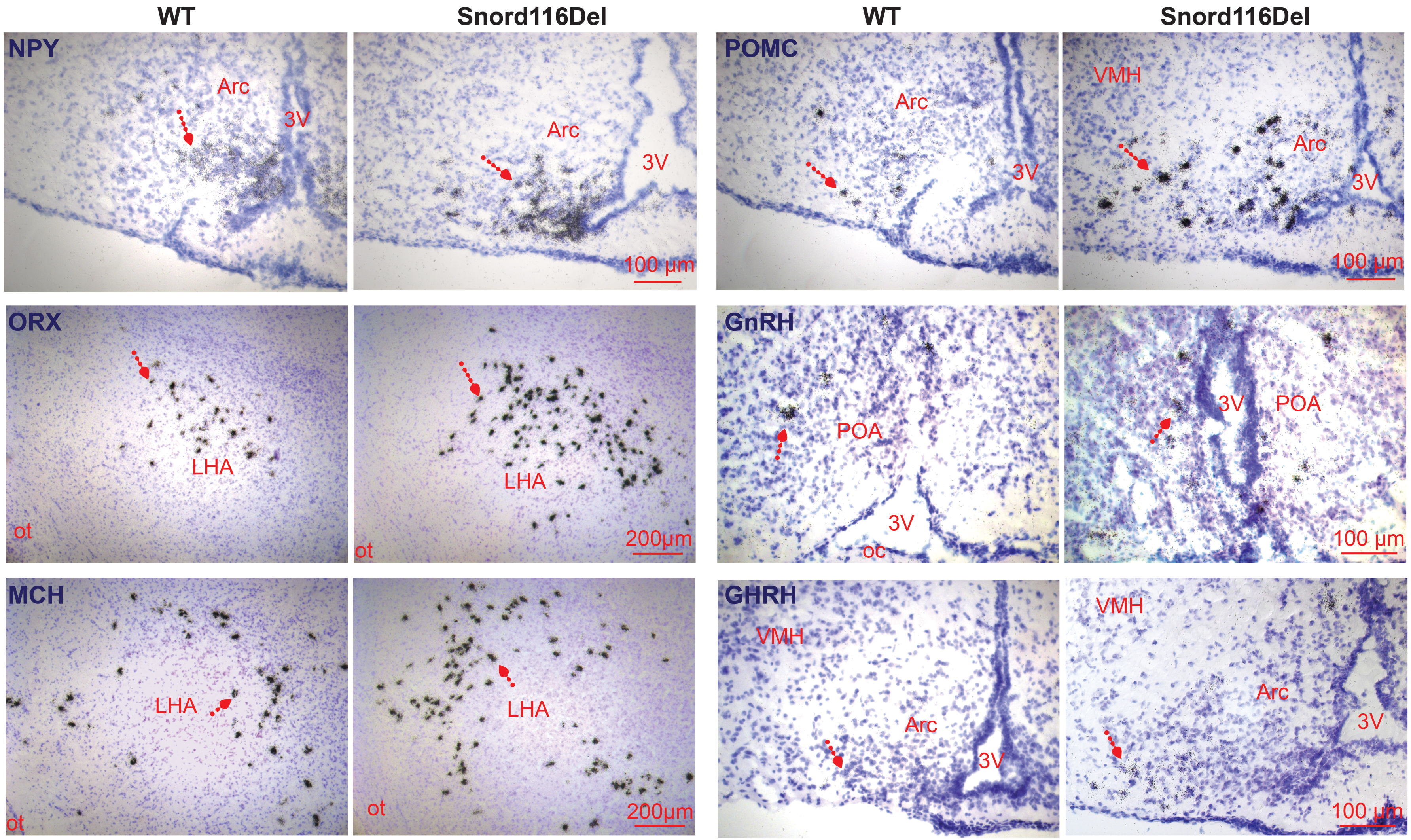

WT
Snord116Del
WT
Snord116Del
NPY
Arc
3V
Arc
3V
100 µm
POMC
Arc
3V
VMH
Arc
3V
100 µm
ORX
LHA
ot
LHA
ot
200µm
GnRH
POA
3V
oc
3V
POA
100 µm
MCH
LHA
ot
LHA
ot
200µm
GHRH
VMH
Arc
3V
VMH
Arc
3V
100 µm

Supplementary table 1:

Gene set enrichment analysis (GSEA) from the collection of gene sets derived from the Biological Process Ontology revealed that 463 out of 569 gene sets were down- regulated in KO with 85 gene sets significant at FDR <25% (Table 1). Out of the remaining 106 gene sets which were upregulated in KO, only 1 gene set was significantly enriched at FDR <25% (Table 2).

Name	Size	ES	NES	Nom p-val	FDR q-val
Regulation_of_nucleocytoplasmic_transport	21	0.657	1.956	0.000	0.068
Regulation_of_intracellular_transport	24	0.610	1.923	0.000	0.069
**Feeding_behavior**	24	0.594	1.915	0.000	0.046
Negative_regulation_of_transport	19	0.589	1.841	0.000	0.066
Cytokine_production	66	0.477	1.825	0.000	0.062
Carbohydrate_transport	18	0.647	1.824	0.000	0.052
Regulation_of_cytokine_production	23	0.586	1.778	0.000	0.090
Fatty_acid_metabolic_process	60	0.481	1.776	0.000	0.079
Positive_regulation_of_transport	21	0.590	1.771	0.015	0.078
Positive_regulation_of_cell_differentiation	24	0.563	1.767	0.000	0.071
Regulation_of_myeloid_cell_differentiation	17	0.598	1.753	0.014	0.078
**Skeletal_development**	97	0.426	1.745	0.000	0.079
Rna_processing	152	0.386	1.736	0.000	0.086
Cellular_defense_response	45	0.494	1.730	0.000	0.089
Positive_regulation_of_multicellular_organismal_process	61	0.437	1.725	0.000	0.091
Protein_targeting	102	0.407	1.722	0.000	0.090
Meiosis_i	20	0.581	1.714	0.000	0.091
Regulation_of_protein_import_into_nucleus	15	0.638	1.713	0.000	0.086
Protein_import_into_nucleus	44	0.480	1.709	0.000	0.087
Regulation_of_transport	64	0.445	1.707	0.000	0.084
Establishment_of_protein_localization	178	0.381	1.706	0.000	0.080
Regulation_of_gene_expression_epigenetic	29	0.521	1.704	0.000	0.079
Meiotic_cell_cycle	34	0.502	1.702	0.014	0.078
Coenzyme_metabolic_process	37	0.487	1.697	0.000	0.078
Nuclear_transport	81	0.414	1.685	0.000	0.085
Nucleocytoplasmic_transport	80	0.417	1.684	0.000	0.083

Protein_localization	201	0.366	1.680	0.000	0.080
Regulation_of_multicellular_organismal_process	140	0.389	1.678	0.000	0.079
Innate_immune_response	18	0.601	1.675	0.018	0.079
Nuclear_import	46	0.472	1.669	0.000	0.079
Activation_of_immune_response	17	0.558	1.667	0.016	0.078
Dna_recombination	46	0.441	1.666	0.000	0.076
Regulation_of_cell_differentiation	57	0.444	1.663	0.000	0.075
Reproductive_process	136	0.389	1.649	0.000	0.086
Protein_import	58	0.442	1.649	0.000	0.084
Icosanoid_metabolic_process	15	0.599	1.646	0.000	0.083
Lipid_metabolic_process	299	0.353	1.645	0.000	0.082
Protein_secretion	29	0.510	1.644	0.042	0.081
Monocarboxylic_acid_metabolic_process	83	0.417	1.642	0.000	0.080
Organic_acid_metabolic_process	171	0.367	1.618	0.000	0.103
Regulation_of_immune_response	32	0.497	1.617	0.000	0.103
Cofactor_metabolic_process	52	0.449	1.614	0.000	0.103
Carboxylic_acid_metabolic_process	169	0.371	1.612	0.000	0.103
Regulation_of_protein_secretion	19	0.543	1.611	0.017	0.101
Immune_effector_process	32	0.490	1.598	0.000	0.112
Macromolecule_localization	221	0.364	1.598	0.000	0.109
Regulation_of_immune_system_process	61	0.421	1.589	0.000	0.115
Intracellular_protein_transport	136	0.377	1.578	0.000	0.126
Regulation_of_response_to_stimulus	54	0.437	1.577	0.000	0.125
Cytokine_secretion	15	0.578	1.562	0.016	0.141
Bone_remodeling	26	0.474	1.553	0.031	0.149
Protein_transport	147	0.356	1.546	0.000	0.153
Adaptive_immune_response_go_0002460	23	0.473	1.537	0.015	0.163
Multi_organism_process	131	0.359	1.531	0.000	0.168
Sensory_perception	181	0.345	1.529	0.000	0.167
Cation_homeostasis	96	0.355	1.527	0.000	0.167
Tissue_remodeling	27	0.481	1.522	0.030	0.171
Female_pregnancy	38	0.425	1.521	0.015	0.169
Positive_regulation_of_immune_response	28	0.488	1.514	0.066	0.173
Regulation_of_binding	57	0.405	1.511	0.013	0.174
Protein_homooligomerization	21	0.508	1.503	0.076	0.182
Immune_system_development	76	0.378	1.501	0.000	0.182

Defense_response	224	0.322	1.487	0.012	0.198
Tissue_development	130	0.349	1.486	0.000	0.198
Viral_reproduction	37	0.431	1.478	0.015	0.210
Meiotic_recombination	17	0.532	1.475	0.045	0.211
Homophilic_cell_adhesion	16	0.534	1.474	0.046	0.211
Negative_regulation_of_multicellular_organismal_process	29	0.468	1.471	0.043	0.213
Positive_regulation_of_immune_system_process	47	0.408	1.470	0.039	0.211
Rna_splicing	80	0.374	1.469	0.000	0.210
Adaptive_immune_response	24	0.481	1.469	0.057	0.208
Amino_acid_metabolic_process	74	0.367	1.469	0.042	0.205
Hemopoietic_or_lymphoid_organ_development	73	0.376	1.468	0.000	0.204
Cellular_lipid_metabolic_process	236	0.325	1.466	0.012	0.204
Hemopoiesis	71	0.373	1.461	0.014	0.210
Inflammatory_response	115	0.344	1.459	0.013	0.210
Dna_repair	121	0.345	1.458	0.024	0.208
Regulation_of_muscle_contraction	18	0.476	1.455	0.063	0.211
Nucleobasenucleoside_and_nucleotide_metabolic_process	50	0.387	1.453	0.081	0.213
Protein_oligomerization	39	0.414	1.452	0.055	0.213
Positive_regulation_of_response_to_stimulus	38	0.421	1.440	0.069	0.228
Immune_response	207	0.319	1.440	0.000	0.226
Positive_regulation_of_caspase_activity	29	0.444	1.438	0.044	0.227
Anatomical_structure_formation	54	0.385	1.437	0.038	0.225

Supplementray Table 2: DNA oligonucleotide sequence of neuropeptides for *in situ* hybridisation

Neuropeptide	DNA oligonucleotide sequence
mouse Snord116	5’-GTTCAGCTTTTCCAAGGAATGTTTGACTGGGAATCATCATAGATCC-3’
mouse GHRH	5′-GCTTGTCCTCTGTCCACATGCTGTCTTCCTGGCGGCTGAGCCTGG-3′
mouse NPY	5′-GAGGGTCAGTCCACACAGCCCCATTCGCTTGTTACCTAGCAT-3′
mouse POMC	5′-TGGCTGCTCTCCAGGCACCAGCTCCACACATCTATGGAGG-3′
mouse MCH	5′-TTTCCTGTGTGGACTCAGCATTCTGAACTCCATTCTCAGCTGG-3′
mouse orexin	5′-CTTTCCCAGAGTCAGGATACCCGCAGCGTGGTTGCCAGCTCCGTGC-3′
mouse GnRH	5′-CAAACACACAGTCAGCAGTAGAATGCCGGCCATCAGTTTGAGGATC-3′
mouse ocytocin	5′- TCCGCGCAGCAGATGCTTGGTCCGAAGCAGCGTCCTTTGCCGC-3′
mouse TH	5′-AACCTTACTCCTCCAGAGGTTCCCTGACCCAGGCTTCCAGTTGTG-3′
